# A Prospective Metagenomic and Metabolomic Analysis of the Impact of Exercise and/or Whey Protein Supplementation on the Gut Microbiome of Sedentary Adults

**DOI:** 10.1128/mSystems.00044-18

**Published:** 2018-04-24

**Authors:** Owen Cronin, Wiley Barton, Peter Skuse, Nicholas C. Penney, Isabel Garcia-Perez, Eileen F. Murphy, Trevor Woods, Helena Nugent, Aine Fanning, Silvia Melgar, Eanna C. Falvey, Elaine Holmes, Paul D. Cotter, Orla O’Sullivan, Michael G. Molloy, Fergus Shanahan

**Affiliations:** a APC Microbiome Ireland, National University of Ireland, Cork, Ireland; b Department of Medicine, National University of Ireland, Cork, Ireland; c Teagasc Food Research Centre, Moorepark, Fermoy, Cork, Ireland; d Section of Biomolecular Medicine, Division of Computational Systems Medicine, Department of Surgery and Cancer, Imperial College London, London, United Kingdom; e Division of Surgery, Department of Surgery and Cancer, Imperial College London, London, United Kingdom; f Alimentary Health Ltd., Cork, Ireland; g Department of Sport and Physical Activity, Human Performance Laboratory, National University of Ireland, Cork, Ireland; h Department of Sports Medicine, Sports Surgery Clinic, Santry, Dublin, Ireland; University of Chicago

**Keywords:** bacteriophages, exercise, metabolism, microbial communities, next-generation sequencing, whey protein

## Abstract

Many components of modern living exert influence on the resident intestinal microbiota of humans with resultant impact on host health. For example, exercise-associated changes in the diversity, composition, and functional profiles of microbial populations in the gut have been described in cross-sectional studies of habitual athletes. However, this relationship is also affected by changes in diet, such as changes in dietary and supplementary protein consumption, that coincide with exercise. To determine whether increasing physical activity and/or increased protein intake modulates gut microbial composition and function, we prospectively challenged healthy but sedentary adults with a short-term exercise regime, with and without concurrent daily whey protein consumption. Metagenomics- and metabolomics-based assessments demonstrated modest changes in gut microbial composition and function following increases in physical activity. Significant changes in the diversity of the gut virome were evident in participants receiving daily whey protein supplementation. Results indicate that improved body composition with exercise is not dependent on major changes in the diversity of microbial populations in the gut. The diverse microbial characteristics previously observed in long-term habitual athletes may be a later response to exercise and fitness improvement.

**IMPORTANCE** The gut microbiota of humans is a critical component of functional development and subsequent health. It is important to understand the lifestyle and dietary factors that affect the gut microbiome and what impact these factors may have. Animal studies suggest that exercise can directly affect the gut microbiota, and elite athletes demonstrate unique beneficial and diverse gut microbiome characteristics. These characteristics are associated with levels of protein consumption and levels of physical activity. The results of this study show that increasing the fitness levels of physically inactive humans leads to modest but detectable changes in gut microbiota characteristics. For the first time, we show that regular whey protein intake leads to significant alterations to the composition of the gut virome.

## Abstract (precis)

The gut microbiota of humans is a critical component of functional development and subsequent health. It is important to understand the lifestyle and dietary factors that affect the gut microbiome and what impact these factors may have. Animal studies suggest that exercise can directly affect the gut microbiota, and elite athletes demonstrate unique beneficial and diverse gut microbiome characteristics. These characteristics are associated with levels of protein consumption and levels of physical activity. The results of this study show that increasing the fitness levels of physically inactive humans leads to modest but detectable changes in gut microbiota characteristics. For the first time, we show that regular whey protein intake leads to significant alterations to the composition of the gut virome.

## INTRODUCTION

Most of the elements of human lifestyle and environment influence the composition or function of the gut microbiome (1, 2). Indeed, the microbiome has been viewed as a transducer of nutrient and other environmental signals for the host (3). Therefore, several investigators have begun to explore whether a sedentary lifestyle or, more specifically, a lifestyle that includes exercise and fitness is associated with changes in the gut microbiota (4–7). This has been assessed in cross-sectional studies of habitual exercisers (8–10) and professional athletes (10–12) in addition to experimental models.

In elite athletes, distinct compositional and functional microbial characteristics, including increased α-diversity, enhanced microbial production of short-chain fatty acids, and greater metabolic capacity, are evident in the gut (11, 12). These microbial features positively correlate with the athletes’ levels of physical activity, in addition to the quantity of dietary protein consumed. In many professional sporting disciplines, as well as amateur sport, intentional protein supplementation (e.g., whey protein) provides a sizeable proportion of athletes’ daily protein intake (12).

Evidence from animal studies highlights the potential for taxonomic manipulation of colonic microbiota following exercise interventions, both with and without concurrent dietary alterations (6, 13, 14). Previously, we have proposed several mechanisms by which exercise and resultant fitness may directly influence the gut microbiota, including effects on gastrointestinal transit time (15), a known driver of the diversity of microbial populations in the gut (16, 17). It appears that physical activity initiated in the juvenile period of development demonstrates a greater potential for fostering a preferential microbiota than exercise commenced in adulthood (6, 18).

However, the relationship between exercise and alterations in the microbiome in humans is compounded by changes in dietary consumption that often accompany physical activity, e.g., increased protein supplement intake.

Building on previous work (11, 12), the present study sought to interrogate correlations between the gut microbiome and levels of physical activity and protein consumption. To do so, using a combination of next-generation shotgun sequencing and metabolomic analysis, we prospectively examined the impact of exercise, with and without whey protein supplementation, on the adult human gut microbiome. We report that 8 weeks of combined aerobic and resistance training led to modest alterations in the composition and activity of the gut microbiome of sedentary individuals. Participants consuming whey protein daily did experience a marked alteration in the diversity of their gut virome following 8 weeks of oral supplementation.

## RESULTS

### Study overview.

**FIG 1:**
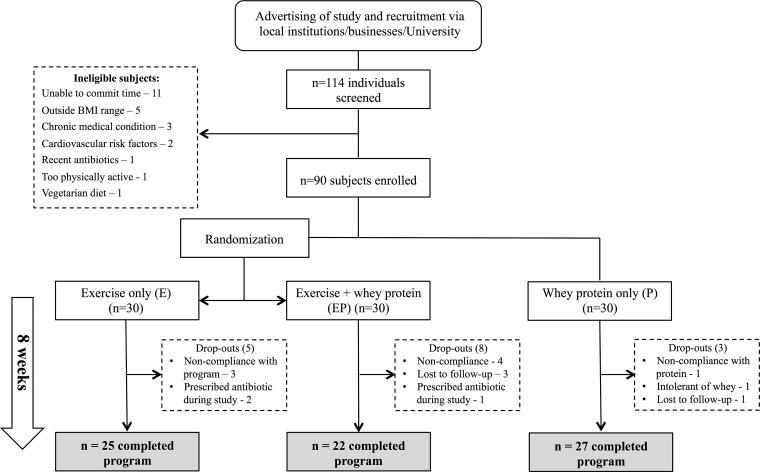
Study design. The figure presents details of study recruitment and allocation of participants to intervention groups as follows: exercise-only group (E), exercise and protein supplementation group (EP), and whey protein supplementation-only group (P). Reasons for volunteer dropout and completion numbers are also outlined. See also Table S1.

Following local advertisement, healthy Irish male and female Caucasian volunteers (*n* = 90) aged 18 to 40 years and with a body mass index (BMI) of between 22 and 35 kg/m^2^ (predominantly overweight or obese) were recruited between January and August 2014 (Fig. 1). The study was conducted in accordance with the Declaration of Helsinki, and, prior to commencement, ethical approval was granted by the Cork Clinical Research Ethics Committee (CREC). All volunteers provided written informed consent. To prospectively measure the effect of *de novo* exercise training on gut microbiota, subjects were required to be physically inactive for at least 3 months prior to study entry (i.e., not engaged in regular structured or unstructured exercise beyond the light physical activities of daily life). All participants were screened for specific exclusion criteria, including regular medication use and history of cardiovascular disease (CVD), diabetes mellitus, or autoimmune disorders (see Table S1 in the supplemental material). Volunteers who had received oral antibiotics or bowel preparations or had suffered gastroenteritis 1 month prior to study enrollment were excluded.

10.1128/mSystems.00044-18.5 TABLE S1  Inclusion and exclusion criteria for study entry. Download TABLE S1, DOCX file, 0.1 MB. Copyright © 2018 Cronin et al. 2018 Cronin et al. This content is distributed under the terms of the Creative Commons Attribution 4.0 International license.

Eligible volunteers were randomized into 2 intervention groups: an exercise-only group (E group) and an exercise plus daily whey protein supplementation group (EP group) (Fig. 1). A separate parallel group consuming whey protein supplementation but not participating in exercise programs (P group) was included in the study as a control. To encourage recruitment to this control group, volunteers were offered an exercise program at a later date, but their participation in the program after the conclusion of the study was not followed extensively. All participants were observed and measured for 8 weeks (*n* = 30 for each group). The exercise-only group (E) participated in an 8-week mixed aerobic and resistance exercise training program. The exercise plus whey protein supplementation group (EP) followed the same exercise program, in addition to consuming the once-daily whey protein supplement. All volunteers were asked to maintain their usual *ad libitum* dietary intake during the intervention period and to refrain from taking additional vitamin, dietary, or herbal supplements.

Participants in the E and EP groups were required to train 3 times per week for 8 weeks. The exercise program consisted of combined aerobic and resistance training. Aerobic exercise was standardized, progressive, and similar in energy expenditure to a "couch-to-5-km-running" program. The intensity of aerobic exercise was moderate, being graded at between 5 and 7 of 10 on the modified Borg rating of perceived exertion (RPE) scale (19). Resistance training consisted of 7 machine-based resistance exercises. Starting weights were calculated at induction at 70% of the individual’s one-repetition maximum (1RM) value. Subjects were required to perform a minimum 3 sets of 8 repetitions. Resistance training was progressive, with aims of increasing resistance weight by 15% to 20% over the 8-week period.

To ensure a uniform and consistent increase in daily protein consumption, subjects in the P and EP arms of the study were required to take a daily 30-g protein supplement containing 24 g of whey protein (donated as an unrestricted grant by Carbery Group, Ballineen, Co. Cork, Ireland). The supplement comprised a blend of whey protein concentrate, isolate, and hydrolyzed whey protein concentrate (see Table S2 for full nutritional details). Subjects’ compliance to daily whey protein supplementation was encouraged using daily text message reminders. Volunteers were required to return empty whey protein sachets to the study site fortnightly before the issuing of further supplement. Subjects with a compliance rate of less than 90% were excluded from the study.

10.1128/mSystems.00044-18.6 TABLE S2  Nutritional content of the daily whey protein supplement (30 g) administered to participants in the EP and P study groups. g, grams; kCal, kilocalories; kJ, kilojoules. Download TABLE S2, DOCX file, 0.04 MB. Copyright © 2018 Cronin et al. 2018 Cronin et al. This content is distributed under the terms of the Creative Commons Attribution 4.0 International license.

Baseline measurements were not significantly different among the three study groups (Table 1).

**TABLE 1:** Baseline demographic and anthropometric characteristics of the study participants with comparisons between the 3 intervention groups^a^

Patient characteristic	Values			*P* value
	Exercise (E) only (*n* = 25)	Exercise + protein (EP) (*n* = 22)	Protein only (P) (*n* = 27)	
Age (yrs)	35 (28, 38)	32 (28, 35)	34 (28, 36)	0.528
No. (%) of females^b^	*n* = 14 (56)	*n* = 12 (55)	*n* = 11 (41)	0.48
Height (cm)	172 (165, 181)	169 (166, 183)	172 (163, 178)	0.67
Weight (kg)	78.8 (70.1, 94.5)	82.3 (69, 98.9)	76.4 (69.8, 87)	0.67
BMI (kg/m^2^)	27.9 (25.1, 29.2)	27.5 (25.7, 30)	27 (24.9, 28.7)	0.761
Resting heart rate (bpm)	72 (65, 81)	68 (61, 79)	74 (66, 78)	0.36
Systolic BP (mm Hg)	128 (117, 134)	125 (121, 136)	125 (118, 130)	0.706
Diastolic BP (mm Hg)	78 (74, 89)	76 (72, 84)	79 (75, 84)	0.543
Waist/hip ratio	0.85 (0.83, 0.89)	0.84 (0.8, 0.93)	0.83 (0.78, 0.88)	0.365
Body fat (%)	32.8 (29, 38.7)	34.7 (29, 37.2)	34.5 (29.3, 39.4)	0.659
Fat mass (kg)	26.3 (22.6, 30.6)	26 (23, 33.1)	26.8 (20.7, 32.9)	0.96
Fat mass (trunk) (kg)	14.1 (10.8, 16.8)	14.1 (11.2, 17.6)	13.7 (9.4, 17.1)	0.878
Lean tissue mass (kg)	52.4 (40.7, 61.4)	51.3 (41.5, 61.5)	47.2 (42.9, 53.3)	0.44
Weekly PA (METS)	462 (298, 1,139)	564 (413, 844)	657 (424, 1,145)	0.599
Weekly PA (kCal)	761 (381, 1,618)	748 (525, 1,127)	762 (512, 1,773)	0.767
Sitting time (h per wk)	56 (40, 61)	62 (47, 76)	51 (33, 62)	0.114
Motorized transport (h per wk)	5 (3.25, 8.3)	3.5 (2, 6)	4.1 (0.8, 7)	0.27

^a^ Values represent medians (interquartile ranges) except where otherwise indicated. *P* values represent results of Kruskal-Wallis tests or chi-square tests. BMI, body mass index; IPAQ, International Physical Activity Questionnaire; METS, metabolic equivalents.

^b^ Data indicate chi-square test results.

Participants were predominantly overweight, with body fat percentages above 30%. There were no significant differences in the participants’ baseline levels of physical activity as assessed using the International Physical Activity Questionnaire (20). All baseline values are expressed as medians and interquartile ranges (IQR).

### Eight weeks of aerobic and resistance training improves body composition and cardiorespiratory fitness profiles in sedentary subjects.

**FIG 2:**
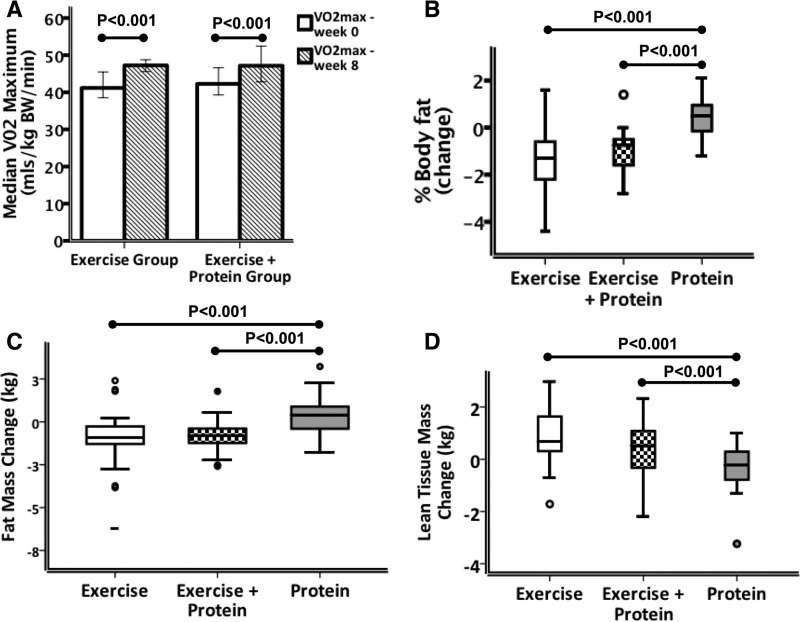
Alterations in cardiorespiratory fitness and body composition following exercise interventions, protein interventions, and combined interventions. (A) Peak aerobic capacity (VO_2max_) per kilogram of body weight as predicted using the Rockport 1-mi walk test was higher in both the E and EP groups following the intervention period, indicating improved levels of cardiorespiratory fitness. Within-group comparisons were tested using the Wilcoxon signed-rank test (*P* < 0.001). (B) Changes in percentages of body fat following the intervention period as measured using DEXA. Percent body fat reduction was significantly greater in the exercise-only group and in the exercise plus protein supplementation group compared to the protein-only group. (C) Absolute changes in body fat mass (in kilograms) following the intervention period demonstrated a significantly greater reduction in both the exercise and exercise plus protein supplementation groups. (D) Absolute change in lean tissue mass (kg), measured using a three-compartment model, indicating significantly greater lean mass accretion in the E and EP groups than in the P group. Error bars represent 95% confidence intervals. See also Tables S3 to S5.

A total of 74 of the 90 participants enrolled in the study completed the 8-week study period (reasons for dropping out are detailed in Fig. 1). At entry, the intervention groups shared similar clinical and anthropometric characteristics. Following the intervention period, both E and EP group participants demonstrated significant and similar improvements in predicted maximal aerobic capacity (VO_2max_) (Fig. 2A). Furthermore, resting heart rate was significantly reduced following the intervention period in both of the exercising groups (E and EP) compared with the protein-only group (*P* = 0.005) (Table S3). Compliance with the prescribed exercise program was high, with a median of 21 sessions (87.5%) performed in both the E and EP groups. The types and levels of exercise training undertaken in both groups were similar, with no statistically significant differences in the aerobic- and resistance-training workloads recorded (Table S4).

10.1128/mSystems.00044-18.7 TABLE S3  Comparison of the postintervention changes (Δ) in clinical and anthropometric variables between groups. Between-group differences in postintervention changes were compared using Kruskal-Wallis tests (*P* values shown; *, *P* < 0.05). When significantly different a Mann-Whitney *U* test was applied to determine between which groups the difference existed. "∞" indicates a difference between the E and P groups; "Ψ" indicates a difference between the EP and P groups (for all data, *P* < 0.05). BMI, body mass index; BP, blood pressure; bpm, beats per minute; weekly PA, self-reported weekly physical activity expenditure assessed using the International Physical Activity Questionnaire; kCals, kilocalories; METS, metabolic equivalents. Download TABLE S3, DOCX file, 0.1 MB. Copyright © 2018 Cronin et al. 2018 Cronin et al. This content is distributed under the terms of the Creative Commons Attribution 4.0 International license.

10.1128/mSystems.00044-18.8 TABLE S4  Measurement of aerobic and resistance training completed in the exercise and exercise plus protein intervention groups (8 weeks), with comparisons of workloads between the two groups. Median values and interquartile ranges (IQRs) are stated in the legend. Comparisons between groups were performed using Mann-Whitney *U* tests. Mins, minutes; Cals, calories expended. Download TABLE S4, DOCX file, 0.1 MB. Copyright © 2018 Cronin et al. 2018 Cronin et al. This content is distributed under the terms of the Creative Commons Attribution 4.0 International license.

In contrast to the protein-only group, the exercise-only group and the exercise plus protein supplementation group experienced significant decreases in percentage body fat, total fat mass, and trunk fat mass during the intervention period (Fig. 2B and C), in addition to an increase in total lean tissue mass (Fig. 2D) (all *P* < 0.001; see also Table S3). Compliance with daily whey protein supplementation in the EP and P groups was high with only one participant excluded due to poor adherence to whey protein supplementation. Whey protein supplementation aside, dietary frequency patterns did not deviate from the volunteers’ usual intake at study entry (Fig. S1). Addition of the 30 g daily whey protein supplement did not favor the EP over E group with respect to body composition improvement; however, the study was not designed to test this hypothesis. No clinically relevant differences in resting-state serum proinflammatory markers were evident following any of the interventions (Fig. S2; see also Table S5).

10.1128/mSystems.00044-18.1 FIG S1  Correspondence analysis of dietary intake of participants. Self-reported dietary information from food frequency questionnaires (FFQ) for all participants was used to perform correspondence analysis of diet at the start and end of the intervention period. The exercise plus protein supplementation group (A and B), the exercise-only group (C and D), and the protein-only group (E and F) maintained diets that remained relatively unchanged during the intervention period. Participants were therefore undifferentiated before and after treatment in relation to diet. Download FIG S1, PDF file, 1 MB. Copyright © 2018 Cronin et al. 2018 Cronin et al. This content is distributed under the terms of the Creative Commons Attribution 4.0 International license.

10.1128/mSystems.00044-18.2 FIG S2  Pre- and postintervention levels of the resting proinflammatory cytokines. Pre- and postintervention distribution of inflammatory cytokines (A) gamma interferon (IFN-γ), (B) IL-6, and (C) TNF-α. Dark central lines represent median values. Bars represent 95% confidence intervals. Outliers are denoted by hollow circles and extreme outliers by asterisks. Download FIG S2, GIF file, 0.01 MB. Copyright © 2018 Cronin et al. 2018 Cronin et al. This content is distributed under the terms of the Creative Commons Attribution 4.0 International license.

10.1128/mSystems.00044-18.9 TABLE S5  Comparison of postintervention changes (Δ) in inflammatory markers between groups. Legend: between-group differences in postintervention changes were compared using the Kruskal-Wallis test (*P* values shown; *, *P* < 0.05). Median changes and interquartile ranges are stated. Where data were significantly different, a Mann-Whitney *U* test was applied to determine between which groups the difference existed. "Ψ" denotes a significant difference between EP and P groups. TNF-α, tumor necrosis factor alpha; IFN-γ, interferon gamma; CRP, C-reactive protein. Download TABLE S5, DOCX file, 0.1 MB. Copyright © 2018 Cronin et al. 2018 Cronin et al. This content is distributed under the terms of the Creative Commons Attribution 4.0 International license.

### Metagenomic assessment of microbiota after exercise and/or dietary adjustment.

**FIG 3:**
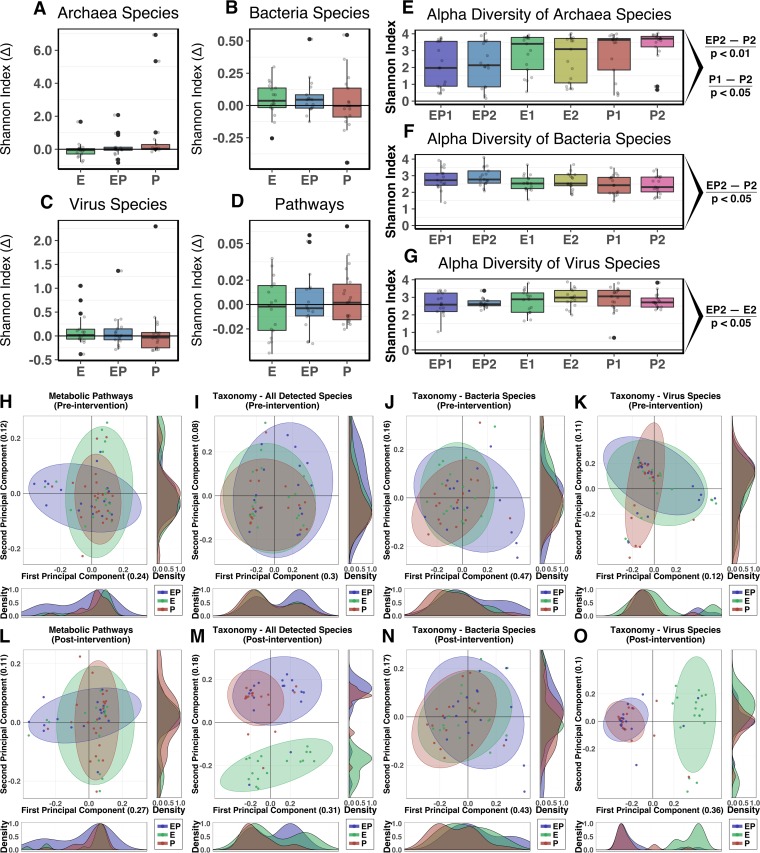
Intervention effects on taxonomic and functional pathway diversity of the intestinal microbiome. (A to D) Percent change (Δ) of Shannon α-diversity H-index values following intervention. No significant variations were presented for taxonomic measurements (A to C) or metabolic pathways (D). (E to G) Pairwise statistical assessment of taxonomy α-diversity demonstrates equal data with respect to the presence of taxonomy between groups at baseline. EP1, combined exercise and protein supplementation group, week 0; EP2, combined exercise and protein supplementation group, week 8; E1, exercise-only group, week 0; E2, exercise-only group, week 8; P1, protein-only group, week 0; P2, protein-only group, week 8. (E) The diversity of *Archaea* was significantly altered after intervention within the P group (*P* < 0.05) and, similarly, was greater in the P group (P2) than in the EP group (EP2) (*P* < 0.01). (F) Postintervention bacterial diversity was greater in the EP group (EP2) in testing against the P group (P2) (*P* < 0.05). (G) Similar levels of virus diversity were presented in the protein supplementation groups (EP and P) following the intervention, with significantly lower diversity in the EP group than in the E group (*P* < 0.05). (H to O) Principal-coordinate analysis (PCoA) of relative abundance profiles for taxonomic and metabolic pathway constructions of the three groups demonstrates the influence of the interventions on the diversity of microbial populations. (H to K) Prior to intervention, group profiles of taxonomic and metabolic pathway diversity were not significantly differentiated. (L to O) Following intervention, a significant separation was identified between the groups for measures of (L) metabolic pathways (*P* = 0.054), (M) all detected species unsegregated by phylogeny (*P* < 0.001), (N) bacteria (*P* < 0.05), and (O) virus species (*P* < 0.001). Specific separations in diversity per intervention group are outlined further in Fig. 4 for virus species and Fig. S3 for all other comparisons. Statistical assessment of PCoA dissimilarity matrices was performed with the Adonis2 permutational multivariate analysis of variance (PERMANOVA) test. (H to O) Density plots were derived from kernel density estimates and scaled to a maximum estimated value of 1 and display concentrations of plotted data along the corresponding plot axis. *P* values were calculated for α-diversity comparisons using the Wilcoxon signed-rank test.

Postintervention alterations (percent Δ) in gut microbial α-diversity did not identify significant modulation in taxonomic composition or metabolic pathways for any of the intervention groups compared to baseline (Fig. 3A to D). A trend of median increase in bacterial diversity was observed for the E and EP groups (Fig. 3B). These findings of moderate alterations of α-diversity were consistent across pairwise comparisons of the groups, with a few notable exceptions. Increased α-diversity of *Archaea* species in the P group following intervention was observed, as was a moderate enhancement of archaeal diversity in the P group compared to the EP group (*P* < 0.05 and *P* < 0.01, respectively; Fig. 3E). After the intervention period, bacterial diversity was greater in the EP group than in the P group (*P* < 0.05; Fig. 3F), while the diversity of virus species was lower in EP group than in the E group (*P* < 0.05; Fig. 3G).

10.1128/mSystems.00044-18.3 FIG S3  Pairwise analysis of microbial taxonomy and metabolic pathways and unsupervised feature detection of pathways. PCoA of metabolic pathways and species-level taxonomy measurement subset according to phylogenetic domain (*Archaea* and *Bacteria*), comparing measurements before and after intervention (time point 1 [TP1] and time point 2 [TP2], respectively). (A to C) Metabolic pathways for the (A) exercise-only group, (B) exercise plus protein supplementation group, and (C) protein-only group. (D to F) *Archaea* species for the (D) exercise-only group, (E) exercise plus protein supplementation group, and (F) protein-only group. (G to I) *Bacteria* species for the (G) exercise-only group, (H) exercise plus protein supplementation group, and (I) protein-only group. (J and K) Cross-validated partial least-squares-discriminant analysis (PLS-DA) of metabolic pathways from all three groups before (J) and after (K) the intervention period identified clusters of pathways related to specific taxa. (J) Prior to treatment, metabolic pathways associated with Prevotella copri were identified in the exercise group (E). (K) After intervention, each group contained a tightly associated cluster of pathways corresponding to either Prevotella copri or Bacteroides vulgatus. The two groups undergoing exercise (E and EP) had separate Prevotella copri-related clusters, while the protein-only-group cluster was composed of Bacteroides vulgatus pathways. Each experimental group was statistically assessed for alterations in diversity resulting from the intervention, with no significant separations detected. Statistical assessment of PCoA dissimilarity matrices was performed with the Adonis2 permutational multivariate analysis of variance (PERMANOVA) test. (A to I) Density plots were derived from kernel density estimates and scaled to a maximum estimated value of 1 and display concentrations of plotted data along the corresponding plot axis. Download FIG S3, PDF file, 2.6 MB. Copyright © 2018 Cronin et al. 2018 Cronin et al. This content is distributed under the terms of the Creative Commons Attribution 4.0 International license.

Principal-coordinate analysis (PCoA) was used to present separation of measures from the taxonomic composition and metabolic pathway models (Fig. 3H to O). Prior to intervention, all 3 groups demonstrated similarity in measures of microbial metabolic pathways and taxonomic β-diversity (Fig. 3H to K). A significant separation between the intervention groups was detected in the Bray-Curtis-derived dissimilarity matrices generated from participants postintervention for metabolic pathways (*P* = 0.054; Fig. 3L), the entirety of detected species (*P* < 0.001; Fig. 3M), and species of bacteria (*P* < 0.05) and viruses (*P* < 0.001) (Fig. 3N and O, respectively). *Archaea* species did not differentiate with intervention (data not presented).

**FIG 4:**
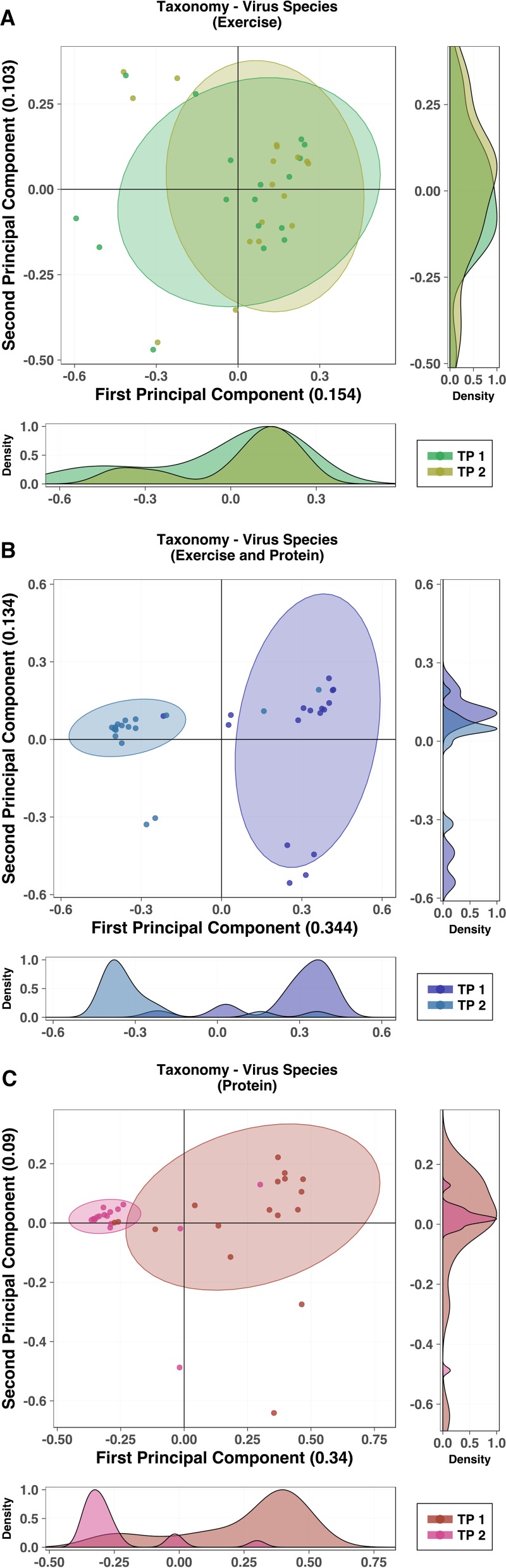
Pairwise analysis of detected virus taxonomy prior to and following intervention. (A to C) PCoA of virus species for each group, comparing virus profiles before and after the intervention period (time point 1 [TP1] [week 0] and time point 2 [TP2] [week 8], respectively). (A) The exercise-only group had virus diversity that was not significantly altered by intervention. (B and C) Diversity of viruses was significantly affected during the intervention period for both groups receiving protein supplementation (*P* < 0.001). The exercise plus protein supplementation group (B) and the protein-only group (C) demonstrated reduced variability of diversity following intervention. Results of pairwise analysis of additional taxonomic and metabolic pathway profiles are presented in Fig. S3. Statistical assessment of PCoA dissimilarity matrices was performed with the Adonis2 PERMANOVA test. (A to C) Density plots were derived from kernel density estimates and scaled to a maximum estimated value of 1 and display concentrations of plotted data along the corresponding plot axis.

Pairwise analysis of taxonomy compared according to high-level phylogeny (*Archaea*, *Bacteria*, and viruses) demonstrated significant alterations of detected virus species in both the EP and P groups following the intervention period that were absent from the exercise-only group (*P* < 0.001; Fig. 4). There were no further significant separations for *Archaea* or *Bacteria* species or for metabolic pathways (Fig. S3A to I). An unsupervised partial-least-squares–discriminant analysis (PLS-DA) approach was used to identify underlying features of the metabolic pathways before and after the intervention period (Fig. S3J and K). Pathways associated with Prevotella copri were shown to cluster with the E group prior to intervention. Following intervention, this cluster was still present but, in addition, separate clusters of P. copri- and Bacteroides vulgatus-associated pathways were apparent within the EP and P groups, respectively. Forty-eight species were detected as being differentially abundant within the three groups (false-discovery rate [FDR] = 0.05). The majority of identified taxa were virus species, predominantly *Lactococcus* phage, within the P and EP groups. No *Archaea* were found to have significantly varied in abundance with treatment in any of the groups (Table S6).

10.1128/mSystems.00044-18.10 TABLE S6  A spreadsheet containing the following combined data: ANCOM results for statistically varied taxa of subjects and for corresponding taxa in whey and control supplements with relative abundance values and a list of significantly varied metabolic pathways and the associated MetaCyc metabolic categories as well as targeted fecal and urinary metabolites with corresponding paired statistical results. Download TABLE S6, XLSX file, 2 MB. Copyright © 2018 Cronin et al. 2018 Cronin et al. This content is distributed under the terms of the Creative Commons Attribution 4.0 International license.

Of the total 23,019 unique (e.g., coenzyme A biosynthesis) and taxonomically specific (e.g., coenzyme A biosynthesis in Akkermansia muciniphila) metabolic pathways included in the metagenomic construction of the models, 619 were identified as having significantly differed among the three intervention groups at either the pretreatment or posttreatment time point (*P* < 0.05). Significantly altered pathways were organized according to a metabolic pathway hierarchy defined by the MetaCyc database and were structured as a heat map of low-level categories of classification (e.g., nucleotide biosynthesis) (Fig. S4). Scaled group means of pathway relative abundances demonstrated modest alterations of microbial metabolic potential. A complete list of the categorized pathways can be found in the supplemental material (Table S6). Further assessment of pathways differing among all groups was performed both within each group (before and after treatment) and between the separate groups. No significant variation within groups was evident following *P* value correction for multiple testing.

10.1128/mSystems.00044-18.4 FIG S4  Categorical heat map of statistically significant metabolic pathways organized into low-level MetaCyc metabolic classifications. (A and B) Metabolic pathways of significantly (*P* < 0.05) varied relative abundances between groups before (A) and after (B) treatment were binned according to the MetaCyc database pathway classification. Scaled group means of pathway relative abundance values demonstrate shifts in the functional potential of the groups following the separate interventions. The Kruskal-Wallis test was used to calculate the *P* values used in the identification of pathways included in metabolic classification. See Table S6 for a complete list of pathways. Download FIG S4, PDF file, 0.1 MB. Copyright © 2018 Cronin et al. 2018 Cronin et al. This content is distributed under the terms of the Creative Commons Attribution 4.0 International license.

Untargeted metabolomic analysis of participant fecal-water and urinary samples revealed no significant separations either within each group pre- and postintervention or between groups at each time point with analysis of the full spectrum of metabolites. Subsequent targeted metabolomic quantification, guided by previous findings (11, 70, 71), revealed significant changes following intervention in the amount of glutamate (fecal water) and *trans*-aconitate (urine) in the protein-only group (*P* < 0.01 and *P* < 0.05, respectively; Table S6). Comparisons of differences (percent Δ) in metabolite quantifications between all groups demonstrated significant variation in the levels of phenylacetylglycine (PAG) and trimethylamine N-oxide (TMAO) (*P* < 0.01 and *P* < 0.05, respectively) in urine, as well as of glutamate (*P* < 0.05) in fecal water, within all groups (Table S6). Such differences were also present in the paired comparisons. Levels of both PAG and TMAO were significantly reduced (Δ = −0.196 and −0.518, respectively) in the E group following the intervention period in comparison to the P group (*P* < 0.05, Table S6).

### Characterization of whey protein supplement microbial content.

Metagenomic sequencing of the whey protein supplement and of a non-dairy-based dietary supplement control revealed a taxonomic profile in the former that was characterized by high proportions of bacteriophage associated with lactic acid bacteria. Notably, these phage were also enriched in participants in receipt of the whey supplement (Table S6). The taxonomic composition of the whey protein and control demonstrated highly divergent microbial contents of the supplements, including taxa detected in participants.

## DISCUSSION

To accurately and consistently increase daily protein intake, we selected a whey protein supplement. Whey protein, a widely used commercial supplement in elite sport and amateur fitness milieu, is known for its muscle accretion effects (21), in addition to its positive influence on energy metabolism (22–24) and, more recently, on appetite control (25). In addition, its use facilitated analysis of the effect of a widely available exercise adjunct on the diversity, composition, and activity of microbial populations in the gut. Somewhat unexpectedly, individuals in the whey protein supplementation-only group (P) experienced a significant alteration in the β-diversity of the gut virome (Fig. 4C). Furthermore, this change was mirrored in the combined exercise and protein supplementation (EP) group (Fig. 4B), suggesting a robust effect of whey protein on the taxonomic richness of the gut virome. To explore this dynamic, a sample of the whey supplement and a sample of a non-dairy-based dietary supplement were sequenced for microbial content. Intriguingly, all bacteriophage and two of the four bacterial species that were significantly altered in the groups receiving whey protein were present in high relative abundance within the whey protein supplement but not the control supplement. Further in-depth experimentation is required to determine whether virus particles from whey protein conclusively transmit to the human gut from consumption and, if so, whether they remain biologically active. However, the overlap in the taxonomic compositions of the whey supplement and participants’ gut microbiome provides a convincing explanation for the source of virome changes observed.

While this examination did not identify a significant impact of short-term combined aerobic and resistance exercise on the diversity of bacterial or archaeal constituents of the gut microbiome, subtle compositional and functional changes were detected in this analysis (Fig. 3 and 4; see also Fig. S3 and S4). Although the results were not statistically significant, the groups engaged in exercise demonstrated less change in archaeal diversity than the protein-only group after the intervention period. In the case of the exercise-only group, a reduction in *Archaea* diversity was observed, suggesting that exercise acts against intestinal *Archaea*. More-extensive investigation is necessary to resolve this issue, but in view of a putative role for *Archaea* in intestinal disorders and as modulators of TMAO concentrations, such an inquiry is justified (26). Changes in *Bacteria* diversity were similarly below the threshold of statistical significance; however, the median differences between groups indicated that those undertaking exercise had increases in bacterial diversity that were absent from the intervention group excluded from exercise. Curiously, the diversity of bacterial species was elevated in the EP group after intervention but the diversity of virus species was uniformly lower. The inverse relationship of these measures is counterintuitive given the predominance of bacteriophage in the detected viruses. However, the influx of such bacteriophage may explain the overall reduction of virus diversity within the group. Furthermore, this increase in the levels of bacteriophage may have been insufficient to profoundly influence the overall diversity of the *Bacteria* due to their selective targeting of only a few bacterial species.

The absence of substantial modulation of the diversity of microbial populations in the gut following the 8-week exercise intervention mirrors recent findings in mice (27). To date, most of the work in humans has focused on elite or professional athletes (10–12) and as a result has explored the relationship between established physical “fitness” and the gut microbiota. Few prospective studies have examined the effect of exercise on the gut microbiota of physically inactive human volunteers (28). The current study is the largest to have done so. It should be acknowledged that the unperturbed adult intestinal microbiome is resilient (29) and may not be subject to significant alteration following an 8-week intervention period. It is likely that the diverse, metabolically favorable intestinal microbiome evident in the elite athlete is the cumulative manifestation of many years of optimized nutrition and of high degrees of physical condition throughout youth and adolescence and during adult participation in professional sports (30). Initial examination of the acute effects of extreme and prolonged endurance exercise, such as in trained military regiments, suggests that prolonged physical stress negatively impacts intestinal permeability and gut microbiota composition (31). However, the results of the present study indicate that exercise at moderate intensity does not exert a deleterious effect on gut microbial composition or function in the untrained subject. Furthermore, the results of this study signify that exercise-induced improvements in cardiorespiratory fitness and body composition are not dependent on substantial alteration of the diversity of microbial populations in the gut. Whether the limited changes in microbiome composition and function detected in this study contributed to the witnessed improvements in body composition and fitness profiles remains unknown.

An intriguing exception to the otherwise minimal differences in metabolomic modification is represented by the controversial metabolite TMAO. Associations between TMAO and cardiovascular disease (CVD) have framed the metabolite as a disease factor; however, high levels have also been observed in populations with low CVD risk (32–35). Elevated levels of TMAO have previously been found in elite athletes (11), and while the presence of TMAO may or may not have deleterious health implications, we demonstrate a potential modulatory effect of exercise on urinary TMAO levels. Participants in the exercise-only group showed levels of urinary TMAO that were reduced below baseline with intervention, while the groups receiving whey protein had increased levels of the metabolite, with the combined-treatment group demonstrating lower levels than the protein-only group. The TMAO precursor phosphatidylcholine comprised less than 0.1% of the constituents of the whey protein supplement used in this study, suggesting a possible direct effect of whey protein and/or exercise on TMAO production. While the data represent a promising paradigm, further work will be necessary to determine the specific mechanisms involved and to rule out unintended dietary influence or influences of host biology (e.g., altered absorption of TMAO with exercise). Additionally, known microbial producers of TMAO (36) were absent from the taxonomic profiling. PAG concentrations were similarly reduced in the exercise-only group, although the metabolite has previously been associated with lean body composition and has been found to be present in increased concentrations in athletes. It has also recently been shown to decrease in urine in thoroughbred racehorses following exercise (37).

Likewise, the data reflecting an increase in the abundance of the P. copri-associated pathways detected in the EP group postintervention supports the work of others which suggested an active role for *Prevotella* species in host metabolic (38, 39) and immune health (40). Studies have linked *Prevotella* with inflammatory and metabolic disorders, including rheumatoid arthritis (41, 42), ankylosing spondylitis (43), and type 2 diabetes mellitus (44). Conversely, and consistent with our findings, increased physical activity has been associated with increases in *Prevotella-*related metabolic pathways in the gut microbiome (10).

It is pertinent to acknowledge the difficulty in controlling all potential confounders of gut microbial composition and activity in this investigation (e.g., diet, wide BMI range). This study attempted to control for the potential impact of dietary variation by instruction of volunteers to maintain their usual *ad libitum* dietary intake. Food frequency questionnaire (FFQ) dietary analysis indicated stability in the volunteers’ dietary patterns; however, like all self-reported methods of dietary intake assessment, FFQ assessment is subject to its limitations, including recall bias (45).

In conclusion, this prospective examination demonstrated that short-to-medium-term combined exercise in healthy, physically inactive adults does not induce drastic alterations in the diversity of bacterial, viral, and archaeal populations in the gut. We highlight an interaction between whey protein intake and the β-diversity of the adult gut virome which requires further exploration. Furthermore, the functional activity of the gut microbiota does not appear to be extensively manipulated by short-term, moderate-intensity exercise and/or whey protein supplementation, although some changes, including alteration of levels of urinary TMAO and PAG excretion, were evident. The alterations in the diversity, composition, and metabolomic profiles of microbial gut populations that we and others have observed in habitual exercisers and professional athletes may represent late responses to exercise or fitness.

## MATERIALS AND METHODS

### Experimental models and subject details.

A description of the human study model used here is outlined in Results under “Study overview.” Male and female volunteers were enrolled. The Cork Clinical Research Ethics Committee (CREC) approved the study before it commenced. Recruitment and assignment of interventions are outlined in detail below.

### Study recruitment and safe participation.

Male and female participants, aged between 18 and 40 years (inclusive), were recruited via online, e-mail, and poster advertisement of the study details. This information was circulated to the study institutions (University College Cork and Cork University Hospital) and local businesses in Cork City, Ireland. Participants were informed that free gymnasium membership would be supplied for the study period. Interested individuals contacted researchers via the study telephone line and were screened initially for inclusion criteria (see Table S1 in the supplemental material). Baseline levels of physical activity were assessed using the International Physical Activity Questionnaire short form (46). If appropriate, a subsequent screening visit at the study site was arranged for further assessment of the exclusion criteria. Safe participation in the exercise program was ensured by medical screening of all participants using an adapted version of the safe participation questionnaire of the American College of Sports Medicine (47).

### Intervention group allocation.

Eligible volunteers were randomized into 2 intervention groups, namely, an exercise-only group (E group) and an exercise plus daily whey protein supplementation group (EP group) (Fig. 1). A separate parallel group consuming whey protein supplementation alone (P group) was included in the study as a control. Participants in the P group were instructed to maintain their usual levels of light physical activity. To encourage recruitment to the control group, volunteers were offered an exercise program at a later date but were not followed extensively during that period. All participants were observed and their responses measured for 8 weeks (*n* = 30 for each group).

### Combined exercise intervention.

Combined aerobic and resistance training was performed at the Mardyke Arena gymnasium at University College Cork, Ireland. All exercise sessions took place at this venue. Volunteers in the P group were asked to maintain their usual levels of physical inactivity for the 8-week period. Participants in the E and EP groups were instructed to adhere to the assigned exercise program and to avoid additional (moderate to vigorous) physical activity outside that prescribed. Participants were instructed to train 3 times per week for the 8-week intervention period. Participants received instructions with respect to the format of the required training program during a 90-min induction session with designated gym instructors. This induction included demonstration of all aerobic and resistance training equipment and the opportunity to ask questions when required. Resistance training machines were customized for individual differences in ranges of motion, and the participants were observed during use of all of the machines, with instructor feedback and correction. For resistance machines, 1-repetition maximum (1RM) values were calculated using standardized methods (48).

The outline of the exercise sessions was as follows. After a 5-min warmup on the treadmill (brisk walking at approximately 4 km/h; modified Borg RPE, 3 to 5/10), participants underwent aerobic training of moderate intensity (modified Borg RPE, 5 to 7/10). To encourage compliance with the prescribed RPE scales, volunteers were reminded of the desired intensities on each of their weekly exercise training programs. The aerobic exercise progressed in duration on a weekly basis but remained of moderate intensity. Initially, aerobic exercise lasted approximately 18 min; by week 8 of the intervention period, the duration of aerobic exercise increased to approximately 32 min depending on the type of aerobic activity chosen by the volunteer. Participants were provided with a choice of aerobic activities, including treadmill jogging/running, use of a cross-trainer device (with no added resistance), use of a stepper machine, and stationary cycling (with mild resistance). The duration for each of these activities was calculated based on the 2011 Compendium of Physical Activities (49) to ensure similar levels of energy expenditure across all activities. To allow variety and to maintain interest, participants were provided with an option of aerobic activities, provided that they did not change exercises within a given training session.

Upon completion of the aerobic exercise activity, participants undertook machine-based resistance training. In summary, participants were required to perform a minimum of 3 sets of 8 repetitions on 7 different resistance machines (3 exercising the upper body, 3 exercising the lower body, and 1 exercising the core muscles). The allowed resistance machine options were as follows: for the upper body, shoulder press, chest press, lateral pulldowns, and seated rowing; for the lower body, leg extension, leg curl, gluteal kick-back, and leg press; for the core muscles, abdominal curls and torso rotation. A minimum limit of 3 sets of 8 repetitions was instituted, with a maximum limit of 3 sets of 12 repetitions. Starting weights were calculated at induction to correspond to 70% of the individual’s one-repetition maximum (1RM) value. Resistance training was progressive, with the aim of increasing the resistance weight by 15% to 20% over an 8-week period. Free-weight use was not permitted.

### Compliance and withdrawal from the study.

Compliance with the prescribed exercise program was monitored remotely by the investigators using a FitLinxx activity monitoring system (Activelinxx, Shelton, CT). All volunteers were provided with a unique identification number for the FitLinxx physical activity recording system and were required to log in and record all activities undertaken at the Mardyke Arena gym during training. Using this tracking system, compliance with the prescribed exercise program was monitored by the investigators. Similarly, the quantity of aerobic and resistance training performed by participants was recorded (Table S4). The FitLinxx software program enables accurate recording of the duration and frequency of training and provides an estimate of energy expenditure during aerobic training. The facility’s FitLinxx software and hardware were subjected to maintenance and recalibration prior to commencement of the study.

Participants noted to have not complied with the exercise regime for more than 7 consecutive days were withdrawn from the study. Individuals requiring antibiotics during the intervention period were also withdrawn from the study, as were participants not complying with whey protein intake requirement in the EP and P groups.

### Measurement visits.

Measurement visits took place at 2 sites: Cork University Hospital and the Mardyke Arena, University College Cork. Baseline measurement was conducted within the 4 days prior to the commencement of the intervention period and once more after the 8-week intervention. Participants were asked to refrain from the use of alcohol and medication and moderate to vigorous physical activity for at least 24 h prior to measurement. To minimize potential effects of diurnal variation, measurement visits took place between 7:00 a.m. and 10:30 a.m. Initially, participants attended the Department of Medicine research facility at Cork University Hospital and sat restfully in a quiet environment. Participants proceeded to participate in measurement of clinical variables, e.g., recording of weight, blood pressure, and heart rate, before undergoing phlebotomy by a trained nurse using universal precautions. Approximately 16 ml of venous blood was withdrawn. Plasma and serum samples were transported immediately to the clinic laboratories at the Mercy University Hospital, Cork. Standardized laboratory techniques were employed for the measurement of hematology and biochemistry indices. Following phlebotomy, individuals underwent a total body dual-energy X-ray absorptiometry (DEXA) scan to assess body composition. When possible, volunteers were asked to provide fresh urine and fecal samples, which were transported at room temperature to Teagasc Moorepark, Fermoy, Co. Cork, where DNA extraction took place. Following completion of the body composition assessment, participants proceeded to the indoor track at the Mardyke Arena gymnasium to undergo a submaximal cardiorespiratory fitness assessment as described below.

### VO_2max_ and body composition measurement.

To prevent injury from unaccustomed vigorous exercise, we chose a submaximal assessment of peak aerobic capacity. Baseline and postintervention levels of cardiorespiratory fitness were measured using a validated submaximal fitness test (50). The Rockport 1-mi walk test was performed in a standardized temperature environment at the indoor running track of the Mardyke Arena. This test was used to estimate maximal oxygen uptake (VO_2max_).

A Lunar iDXA machine (GE Healthcare, Madison, WI) at the Bone Densitometry Unit, Cork University Hospital, was used. Body composition was analyzed using enCORE software (V.13.4, 2010) and a three-compartment (fat mass, bone mass, lean tissue) body composition model. Volunteers were scanned postvoiding and dressed in light clothing, with metal-wear removed where present. Quality control (QC) analysis was performed on the iDXA machine before use of the machine on each measurement day.

### Inflammatory cytokine measurement.

Blood samples (4 ml) from participants were collected in serum separator clot activator blood collection tubes (Greiner Bio-One, Stonehouse, United Kingdom; reference no. 454071). The blood samples were allowed to rest upright on the laboratory bench for 30 min before centrifugation was performed at 1,000 × *g* for 20 min at room temperature. Approximately 2 ml of supernatant sera was then harvested by pipette, frozen, and stored at −80°C in polypropylene cryogenic vials. At a later date, following a complete thaw, resting levels of proinflammatory cytokines were measured using a mesoscale discovery (MSD) platform (Meso Scale Discovery, Rockville, MD). The MSD system is an electrochemiluminescence-based solid-phase multiplex assay. An ultrasensitive human proinflammatory I, V-Plex immunoassay panel containing interleukin-6 (IL-6), IL-8, tumor necrosis factor alpha (TNF-α), IL-10, and gamma interferon was used to measure serum levels. Samples were diluted 1:2 according to the manufacturer’s protocol, and samples from all 3 intervention groups were dispersed across each MSD plate. The lower limit of detection was <1 pg/ml for all assays. All plasma samples were measured in duplicate, and the mean cytokine concentration of the duplicates (in picograms per milliliter) was used for analysis.

### Dietary data collection.

Dietary data were collected by means of a 146-item food frequency questionnaire (FFQ) as outlined previously (12). Participants were asked to record their usual pattern of dietary intake over the previous 8 weeks. The FFQ used was an adapted version of the questionnaire used in the United Kingdom arm of the European Prospective Investigation into Cancer (EPIC) study (51), which was based on the original Willet FFQ (52). Completed FFQs were coded and dietary data were visualized with correspondence analysis using the ade4 package (53) in the R programing environment (V.3.3.2).

### DNA extraction and metagenomic sequencing of fecal microbiome and whey protein supplement.

DNA was extracted from the donated fresh fecal samples received at the Teagasc Moorepark research facility using a QIAmp DNA stool minikit (Qiagen, Crawley, West Sussex, United Kingdom) (54). Samples were provided by participants as partial evacuations into sterile containers and, when not immediately transported for DNA extraction, were held at 4°C for no more than 12 h. Samples were prepared for DNA extraction by manual homogenization of a portion of the sample representing all microenvironments (i.e., core and external surface) of the feces. The provided manufacturer’s protocol was used with modification whereby a zirconia bead (Stratech Scientific) cell disruption bead-beating step (performed three times for 30 s each time) was introduced in order to enhance homogenization of the extraction material. DNA extracts and the remaining fecal samples were subsequently stored at −80°C until they were prepared for sequencing.

Metagenomic libraries were prepared using an Illumina Nextera XT DNA library preparation kit (Illumina Inc., USA) in complete accordance with the manufacturer’s protocol (15031942; Illumina). Library input DNA was normalized to 0.2 ng/µl using fluorometric quantification and Qubit system 2.0 (Thermo, Fisher). Library fragmentation and amplification were performed using a G-Storm GS1 thermal cycler, with subsequent library purification achieved with AMPure magnetic beads (Beckman Coulter, Inc.) at a DNA/AMPure ratio of 1:1.8. Confirmation of the sizes of the library fragments was carried out on an Agilent 2100 Bioanalyzer system, with final assessment of library molar concentration (2 nM) performed using a Roche LightCycler 480 instrument (Roche Applied Science) and a Kapa library quantification kit (Kapa Biosystems). A total of 8 equimolar library pools of samples were made prior to shipping of the pools on dry ice for sequencing on an Illumina Hiseq 2500 (chemistry V.4.0) sequencing platform (Beckman Coulter, Inc.; Genomics Inc., Danvers, MA). High-throughput sequencing was performed using the high-output run mode for 2 × 125-bp paired-end reads with the addition of a PhiX library (1%) to estimate sequence quality. A sample of the whey protein used in the study and a sample of an oat-based nutritional supplement used as a control were both processed in a manner identical to that used with the fecal samples for the extraction of microbial DNA and preparation of metagenomic libraries. Sequencing of the supplement libraries was performed using an Illumina MiSeq (chemistry V.3.0) platform in high-output run mode for 2 × 300-bp paired-end reads (Teagasc sequencing facility).

### Bioinformatic processing of microbial metagenomic sequencing.

Processing of metagenomic FASTQ sequence files proceeded with the removal of human-derived contaminant sequences with NCBI Best Match Tagger (BMTagger) software, while trimming and removal of duplicate reads or of reads of substandard quality were performed with Picard and SAM tools. Functional profiling of high-quality processed reads was facilitated by use of the Human Microbiome Project (HMP) Unified Metabolic Analysis Network (HUMAnN2 V.0.99) pipeline (55). Models of microbial metabolic pathways produced by HUMAnN2 were derived from the MetaCyc database (56) and were the basis for analyses performed on microbial metabolic profiling. Taxonomic profiling was facilitated by use of the Kraken taxonomy assignment software tool (V.0.10.6) (57).

### Metabolomic sample preparation.

Samples were stored at −80°C prior to analysis. Urine samples were subjected to vortex mixing and then centrifuged at 1,600 × *g* for 10 min to remove precipitated proteins and particulates. For metabolic profiling analysis by reversed-phase (RP) and hydrophilic interaction chromatography (HILIC) ultraperformance liquid chromatography-mass spectrometry (UPLC-MS), samples were prepared as follows: 200 µl of supernatant was diluted (1:1) with high-purity (ultraperformance liquid chromatography [HPLC]-grade) water, subjected to vortex mixing, centrifuged at 2,700 × *g* for 20 min, and divided into aliquots for analysis. Quality control (QC) samples were prepared by pooling 50-µl volumes of each sample. For ^1^H nuclear magnetic resonance (^1^H-NMR) spectroscopy, each sample contained 540 µl of urine mixed with 60 µl of phosphate buffer (pH 7.4; 80% D_2_O) containing a 1 mM concentration of the internal standard, 3-(trimethylsilyl)-[2,2,3,3,-2H4]-propionic acid (TSP)–2 mM sodium azide (Na^3^N), as described previously (58). During the analyses, samples were maintained at 4°C in the autosampler.

Fecal samples underwent two freeze-thaw cycles. Following the freeze-thaw cycles, 100 mg of homogenized sample was placed in a microtube containing 250 µl of 25% acetonitrile (ACN) (1:2 ACN/H_2_O), 2 mM sodium azide, and ~0.05 g 1-mm-diameter zirconia beads. Each microtube was processed for 10 s in a Biospec bead beater. Samples were then centrifuged at 16,000 × *g* for 20 min. The fecal-water supernatant was subsequently centrifuged through centrifuge tube filters (cellulose acetate membrane; pore size, 0.22 µm) to remove any remaining particulate matter. The centrifuge tube filters were washed three times with 25% acetonitrile prior to use. The resulting fecal water was prepared for UPLC-MS profiling using HILIC by diluting 3:1 with acetonitrile and for bile acid profiling by diluting 1:1 with isopropanol. Samples were subjected to vortex mixing and incubated at −20°C for 1 h. Following the incubation step, samples were centrifuged at 4°C at 16,000 × *g* for 1 h and divided into aliquots for analysis. QC samples were prepared by pooling 20-µl volumes of each fecal-water sample followed by preparation as described above. For ^1^H-NMR spectroscopy, 50 µl of the filtered fecal water was added to a glass tube (Pyrex), which was placed under a nitrogen gas flow for 30 min or until all the liquid had evaporated. The dried sample was reconstituted with 540 µl of D_2_O and 60 µl of phosphate buffer solution as described above. The solution was mixed and sonicated for 5 min before undergoing further centrifugation at 14,000 rpm for 10 min, and then 600 µl of the supernatant was transferred to an NMR tube for ^1^H-NMR spectral acquisition.

### Metabolomic analysis.

RP, HILIC, and bile acid UPLC-MS metabolic profiling experiments were performed using a Waters Acquity Ultra Performance LC system (Waters, Milford, MA) coupled to a Xevo G2 quadrupole-time of flight (Q-TOF) mass spectrometer (Waters, Milford, MA) with an electrospray source. Samples were analyzed in randomized order, with QC analyzes performed every 10 samples. First, urine samples were analyzed using UPLC-MS and an RP chromatographic method with both positive and negative MS ionization modes. Second, to separate and detect the more polar molecules, a HILIC chromatographic stage was used with the positive MS ionization mode. Fecal-water samples underwent analysis using HILIC and bile acid profiling chromatographic methods in positive and negative ionization modes, respectively. HILIC, RP, and bile acid profiling liquid chromatographic separation procedures were performed as previously described (59, 60). Mass spectrometry was performed with the following settings. Capillary and cone voltages were set at 1.5 kV and 30 V, respectively. The desolvation gas level was set at 1,000 liters/h at a temperature of 600°C. The cone gas level was set to 50 liters/h. The source temperature was set to 120°C. To ensure the accuracy of the mass data, a lock-spray interface was used, with leucine enkephalin {556.27741 Da ([M+H]+), 554.2615 Da ([M−H]−)} solution used as the lock mass at a concentration of 2,000 ng/ml and a flow rate of 15 µl/min.

^1^H-NMR spectroscopy was performed on the aqueous-phase extracts at 300 K on a Bruker 600-MHz spectrometer (Bruker Biospin, Germany) using a standard one-dimensional (1D) pulse sequence corresponding to RD − *g*_z1_ − 90° − *t*_1_ −90° − *t*_*m*_ − *g*_z2_ − 90°− ACQ (58), where the value of 90° represents the applied 90° radio frequency pulse; the relaxation delay (RD) was set at 4 s, the interpulse delay (*t*_1_) was set at 4 µs, the mixing time (*t*_*m*_) was set at 10 ms, the magnetic field gradients (*g*_z1_ and *g*_z2_) were applied for 1 ms, and the acquisition period (AQA) was 2.7 s. Water suppression was achieved through irradiation of the water signal during RD and *t*_*m*_. Urine sample spectra were acquired using 4 dummy scans followed by 32 scans whereas fecal spectra were acquired using 256 scans and 4 dummy scans and collected into 64 K data points. A spectral width of 12,000 Hz was used for all the samples. Prior to Fourier transformation, the free induction decay (FID) values were multiplied by an exponential function corresponding to a line broadening of 0.3 Hz.

### Metabolomic data treatment.

The raw mass spectrometric data acquired were preprocessed using xcms in R. centWave peak picking methods were used to detect chromatographic peaks (61). The xcms-centWave parameters were data set specific. Feature grouping across samples was performed using the “nearest” method within xcms. Peak filling and MinFrac (0.5), and coefficient of variation (CV) (0.3) filters were applied to the features. Data were normalized using median fold change normalization to the median data set (62).

^1^H-NMR spectra were automatically corrected for phase and baseline distortions and referenced to the TSP singlet at δ 0.0 using TopSpin 3.1 software. Spectra were then digitized into 20 K data points at a resolution of 0.0005 ppm using an in-house MatLab R2014a (MathWorks, Inc.) script. Subsequently, spectral regions corresponding to the internal standard (δ −0.5 to 0.5) and water (δ 4.6 to 5) peaks were removed. In addition, urea spectra (δ 5.4 to 6.3) were removed from the urinary spectra. Spectra were normalized using median fold change normalization to the median spectrum (62). Combinations of data-driven strategies, such as SubseT optimization by reference matching (STORM) (63) and Statistical TOtal Correlation SpectroscopY (STOCSY) (64), and analytical identification strategies were used to identify metabolites of interest from ^1^H-NMR data sets. Specifically, a catalogue of 1D ^1^H-NMR and 2D NMR experiments was performed using techniques such as J-RESolved spectroscopy, ^1^H-^1^H TOtal Correlation SpectroscopY (TOCSY), ^1^H-^1^H COrrelation SpectroscopY (COSY), ^1^H-^13^C Hetero-nuclear Single Quantum Coherence (HSQC), and ^1^H-^13^C Heteronuclear Multiple-Bond Correlation (HMBC) spectroscopy. Finally, for those metabolites giving ambiguous data, e.g., TMAO, the metabolites were confirmed using *in situ* spiking experiments and authentic chemical standards. Semiquantification data corresponding to the identified metabolites were calculated through peak intensity measurements of the normalized ^1^H-NMR spectra using an in-house script. GC-MS data were processed using MassHunter Quantitative Analysis (Agilent Technologies) software.

### Quantification and statistical analysis.

Statistical analysis was carried out using the Statistical Package for the Social Sciences V.23 (SPSS, Inc., Chicago, Illinois) and the R statistical programing environment (V.3.3.2). Due to the predominance of non-normally distributed data, nonparametric analyses were performed to compare baseline clinical and demographic variables between groups. Similarly, nonparametric statistical tests were employed in the analysis of microbiome and metabolomics data. Clinical data are presented as medians and interquartile ranges (IQR), unless stated otherwise. Between-group differences in baseline, follow-up, and postintervention changes (Δ) in clinical and demographic data were compared using the Kruskal-Wallis test. For significantly different results, a Mann-Whitney *U* test was performed to determine the groups between which this difference applied. Where stated, the Wilcoxon signed-rank test was used to compare baseline and postintervention values within intervention groups. A type I error rate of ≤0.05 was considered significant in all cases. Correction of *P* values relating to microbiome and metabolomic analysis was performed using the Benjamini-Hochberg false-discovery rate (FDR) (65) in the base *stats* package in R. Statistical assessment of dissimilarity matrices (Bray-Curtis) derived from microbial data was facilitated with the adonis2 function in the vegan R package (V.2.4-3) (66). Identification of statistically relevant taxonomic features was performed with the analysis of composition of microbiomes (ANCOM) test as implemented in the R package of the same name (V.1.1-3) (67). Detection of underlying features of metabolic pathways was performed with unsupervised cross-validated partial-least-squares–discriminant analysis (PLS-DA) and the KODAMA algorithm from the R package of the same name (V.1.4) (68). Measurements of alpha diversity and calculations of relative abundances were also performed with the vegan package. Relative-abundance data were generated separately for identified species within each phylogenetic domain (e.g., *Bacteria*).

For metabolomic analysis, the resulting ^1^H-NMR and LC-MS data sets were imported into MatLab to conduct multivariate statistical analysis. Data were centered and scaled to account for the repeated-measures design and then modeled using partial-least-squares–discriminant analysis (PLS-DA) with Monte Carlo cross-validation (MCCV) (69). The fit and predictability of the models obtained were determined and expressed as R2 and Q2 values, respectively.

### Data availability.

The microbial DNA sequences have been deposited in the European Nucleotide Database (ENA) database under ID code PRJEB20054.
