# Supplementary material for: A Prospective Metagenomic and Metabolomic Analysis of the Impact of Exercise and/or Whey Protein Supplementation on the Gut Microbiome of Sedentary Adults
Source: mSystems. 2018 Apr 24;3(3):e00044-18. doi: 10.1128/mSystems.00044-18 (PMC5915698; doi:10.1128/mSystems.00044-18)
Supplement: TABLE S1 [file sys003182228st1.docx]

| **Inclusion Criteria** |
| --- |
| - Low physical activity level as per the International Physical Activity Questionnaire (Short form) - Not currently or recently (last 3 months) involved in regular or organized amateur sport or exercise - Non-smoker - Not on regular medications - Aged 18 to 40 - Body Mass Index between 22 to 35 (inclusive) |
| **Exclusion Criteria** |
| - Personal history of coronary artery disease, congenital heart disease or any cardiovascular disease - Family history of known coronary artery disease before 45 years of age - Uncontrolled hypertension (>140/90 mmHg) - Known renal or hepatic impairment - Type 1 or type 2 diabetes mellitus - Pulmonary disease – not including well-controlled, mild asthma - Primary or secondary immunodeficiency or autoimmune disorder - Current smoker or ex-smoker of less than 3 months duration - Psychiatric disorders including previous history of depression - A history of substance abuse - Current or recent involvement in another clinical research study - Known or suspected hypersensitivity to the dietary supplementation - Gastro-intestinal disease e.g. coeliac disease, inflammatory bowel disease, Irritable Bowel Syndrome - Previous significant gastro-intestinal surgery e.g. Total colectomy - Suspected or confirmed pregnancy |
