# Supplementary material for: A Prospective Metagenomic and Metabolomic Analysis of the Impact of Exercise and/or Whey Protein Supplementation on the Gut Microbiome of Sedentary Adults
Source: mSystems. 2018 Apr 24;3(3):e00044-18. doi: 10.1128/mSystems.00044-18 (PMC5915698; doi:10.1128/mSystems.00044-18)
Supplement: TABLE S2 [file sys003182228st2.docx]

| **Nutritional Information** | **Typical per 30g serving** | **Typical per 100g serving** |
| --- | --- | --- |
| Energy | 125 kcal/521kJ | 416 kcal/1743kJ |
| Protein | 24 g | 80 g |
| Carbohydrate  *of which sugars* | 1.5 g  0.9 g | 5.0 g  3.0 g |
| Fat  *of which saturates* | 2.5 g  1.8 g | 8.2 g  6.0 g |
| Dietary fiber | 0.08 g | 0.26 g |
| Salt | 0.12 g | 0.42 g |
