# Supplementary material for: A Prospective Metagenomic and Metabolomic Analysis of the Impact of Exercise and/or Whey Protein Supplementation on the Gut Microbiome of Sedentary Adults
Source: mSystems. 2018 Apr 24;3(3):e00044-18. doi: 10.1128/mSystems.00044-18 (PMC5915698; doi:10.1128/mSystems.00044-18)
Supplement: TABLE S3 [file sys003182228st3.docx]

|  | **Exercise (E) Group (n=25)** | **Exercise + Protein (EP) Group (n=22)** | **Protein only (P) Group**  **(n=27)** | **p-value** |
| --- | --- | --- | --- | --- |
| Weight (kg) | -0.9 (-2.6, 0.9) | -0.8 (-1.6, 0.1) | -0.5 (-1.3, 0.6) | 0.549 |
| BMI (kg/m^2^) | -0.3 (-0.9, 0.2) | -0.2 (-0.6, 0) | -1.1 (-0.4, 0.2) | 0.419 |
| Resting heart rate (BPM) | -5 (-16, 6) ^∞^ | -5 (-9, 3) ^Ψ^ | 4 (-3, 10) | 0.005* |
| Systolic BP (mmHg) | -8 (-12, 1) | -8 (-16, 0) | -4 (-11, 0) | 0.545 |
| Diastolic BP (mmHg) | -5 (-12, 1) | -6 (-9, -2) | -5 (-8, 0) | 0.785 |
| Waist:Hip ratio | -0.01  (-0.03, 0.01) | -0.02  (-0.04, 0.01) | 0  (-0.01, 0.04) | 0.07 |
| Body fat (%) | -1.3 (-2.4, -0.5) ^∞^ | -0.8 (-1.7, -0.5) ^Ψ^ | 0.5 (-0.2, 1) | <0.001* |
| Fat mass (kg) | -0.9 (-1.5, -2.7) ^∞^ | -0.8 (-1.2, -0.4) ^Ψ^ | 0.4 (-0.5, 0.9) | <0.001* |
| Fat mass (trunk) (kg) | -0.5 (-1, 02) ^∞^ | -0.6 (-0.8, -1) ^Ψ^ | 0.1 (-0.4, 0.6) | 0.001* |
| Lean tissue mass (kg) | 0.7 (0.3, 1.8) ^∞^ | 0.5 (-0.4, 1.1) ^Ψ^ | -0.2 (-0.9, 0.3) | 0.001* |
| Weekly PA (METS) | 1,159  (712, 1,964) ^∞^ | 1,265  (434, 2,487) ^Ψ^ | 111  (-244, 634) | <0.001* |
| Weekly PA (kCals) | 1,442  (818, 2,628) ^∞^ | 1,789  (571, 3,289) ^Ψ^ | 184  (-418, 800) | <0.001* |
| Sitting time (hours per week) | -5 (-17, 2) | -12 (-30, 1) | -5 (-18, 1) | 0.407 |
| Motorized transport (hours per week) | 0 (-3.3, 2.8) | 0 (-1, 1.3) | 0.1 (-0.4, 5) | 0.519 |
