# Supplementary material for: A Prospective Metagenomic and Metabolomic Analysis of the Impact of Exercise and/or Whey Protein Supplementation on the Gut Microbiome of Sedentary Adults
Source: mSystems. 2018 Apr 24;3(3):e00044-18. doi: 10.1128/mSystems.00044-18 (PMC5915698; doi:10.1128/mSystems.00044-18)
Supplement: TABLE S4 [file sys003182228st4.docx]

| **Workload parameter** | **Exercise Group (E) (n=25)** | **Exercise + Protein Group (EP) (n=22)** | **p-value** |
| --- | --- | --- | --- |
| Number of exercise sessions attended | 21 (16, 23) | 21 (20, 23) | 0.317 |
| Duration of aerobic training (mins) | 671 (436, 728) | 625 (539, 685) | 0.983 |
| Aerobic exercise energy expenditure (Cals) | 6,043 (3754, 7411) | 5,869 (5291, 7324) | 0.654 |
| Calories expended per body weight (Cals/kg) | 69 (47, 90) | 74 (62, 83) | 0.685 |
| Total number of repetitions | 4,861 (4273, 6008) | 4,874 (4281, 6136) | 0.685 |
| Total weight lifted (Tonnes) | 171.6  (136.8, 205) | 169.7  (111, 238.4) | 0.701 |
| Total weight lifted (kg) per kg of body weight | 1,962 (1753, 2282) | 2,288 (1464, 2746) | 0.43 |
