# Supplementary figures and images for: A Prospective Metagenomic and Metabolomic Analysis of the Impact of Exercise and/or Whey Protein Supplementation on the Gut Microbiome of Sedentary Adults
Source: mSystems. 2018 Apr 24;3(3):e00044-18. doi: 10.1128/mSystems.00044-18 (PMC5915698; doi:10.1128/mSystems.00044-18)

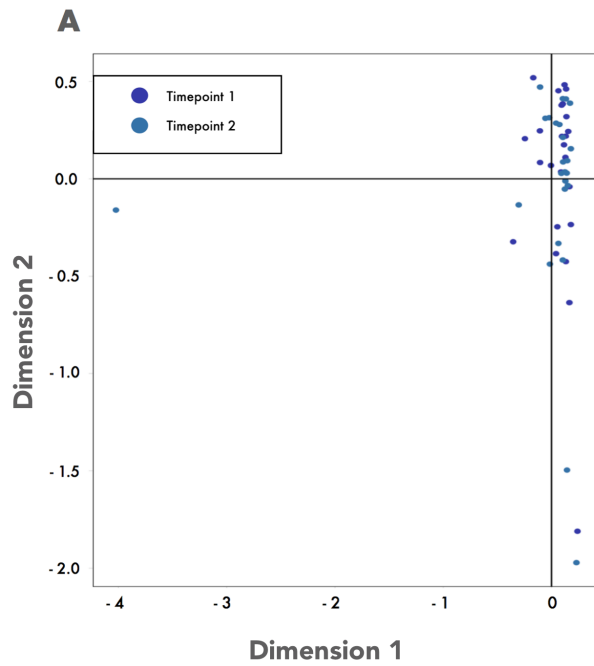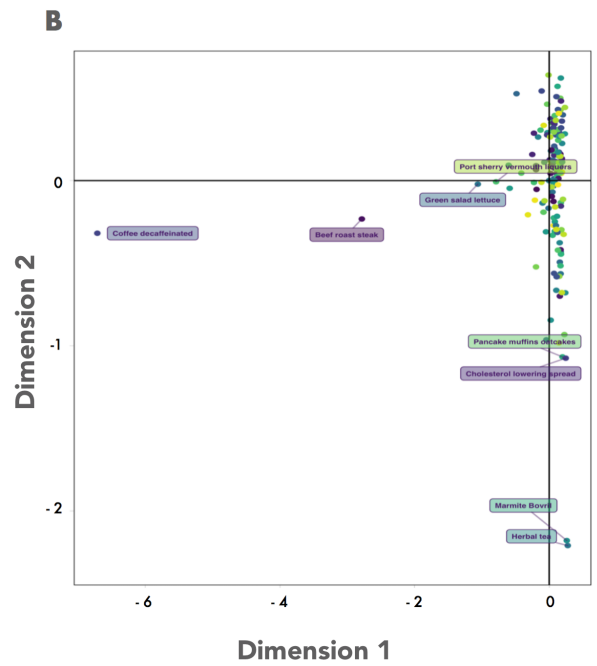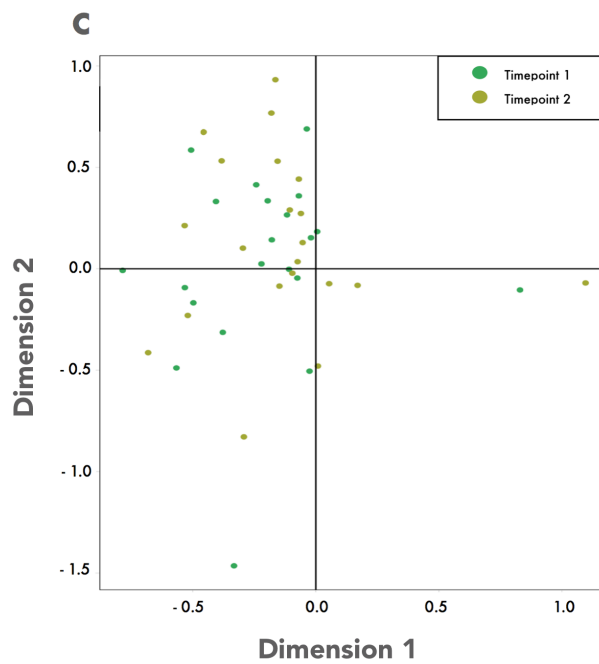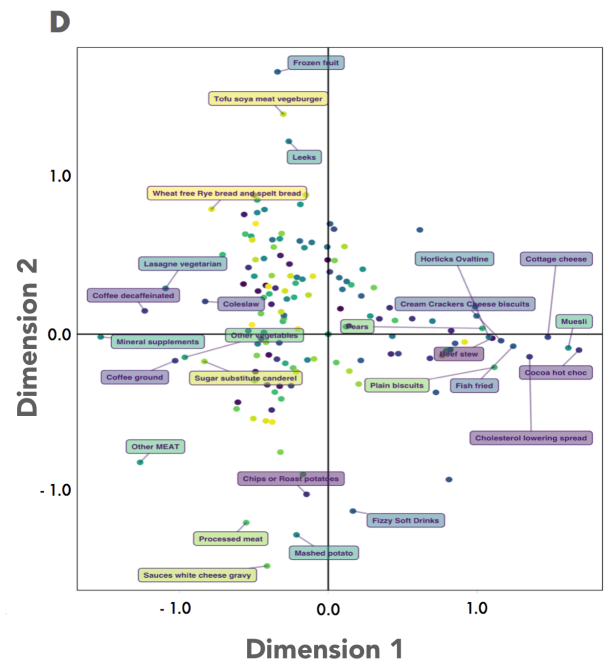

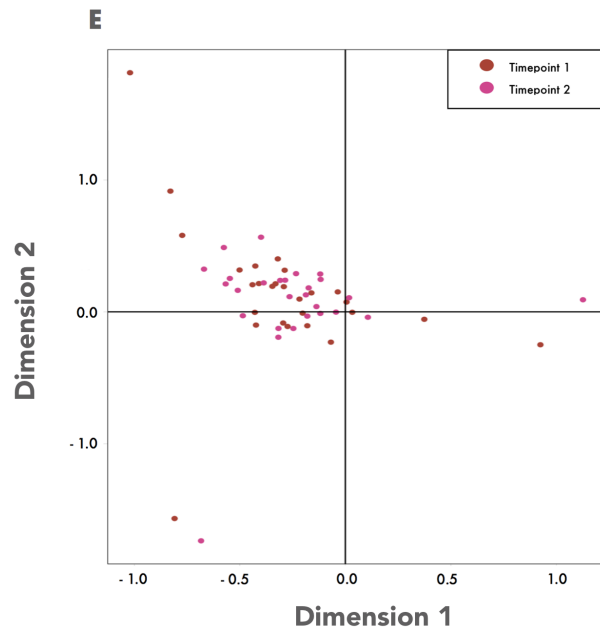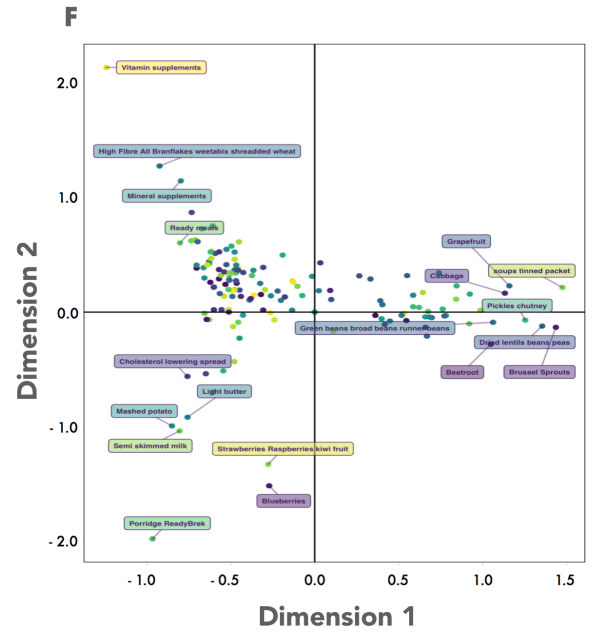

Supplement: FIG S1 [file sys003182228sf1.pdf]

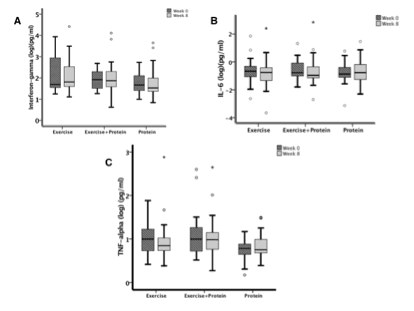

Supplement: FIG S2 [file sys003182228sf2.gif]

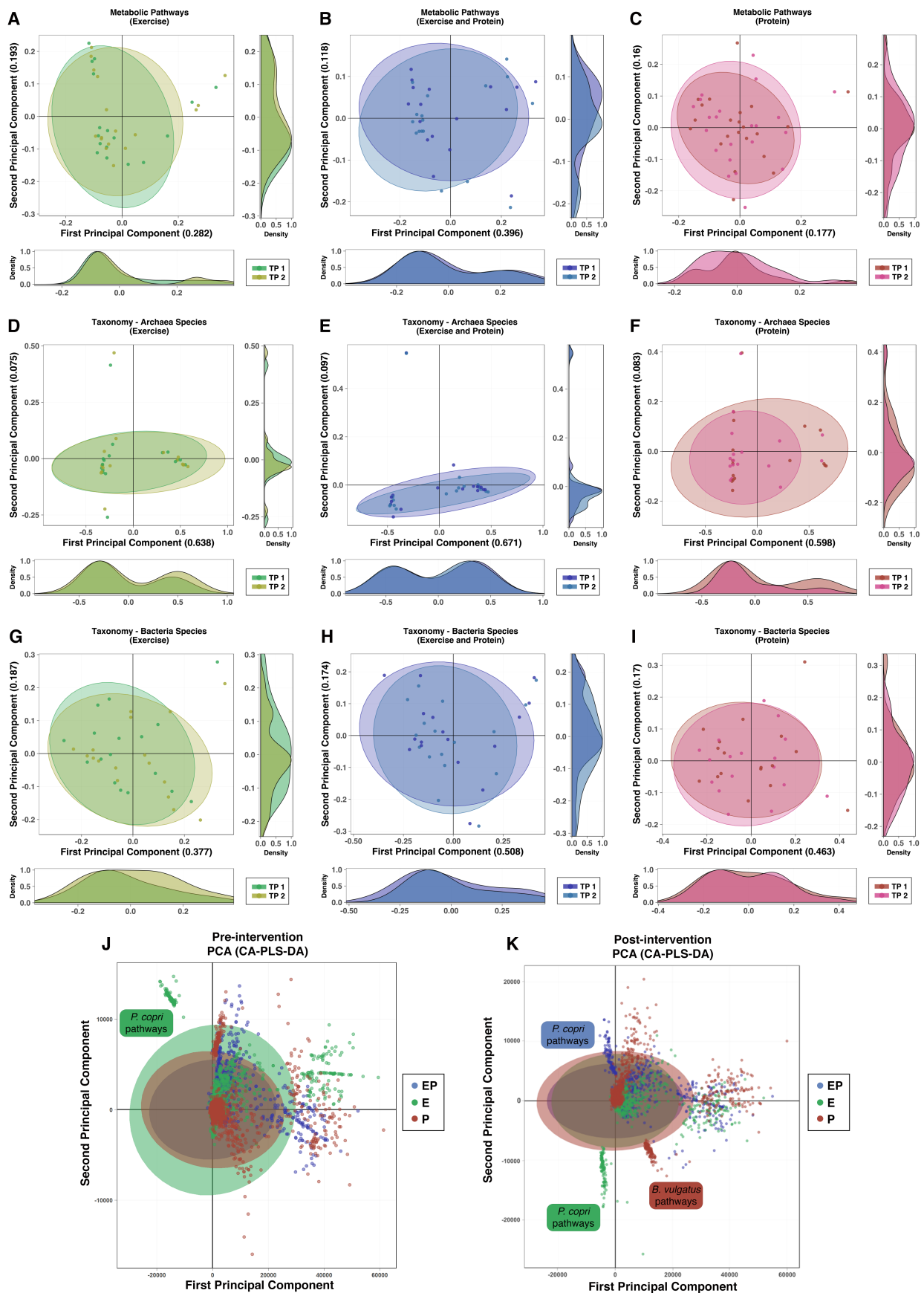

Supplement: FIG S3 [file sys003182228sf3.pdf]

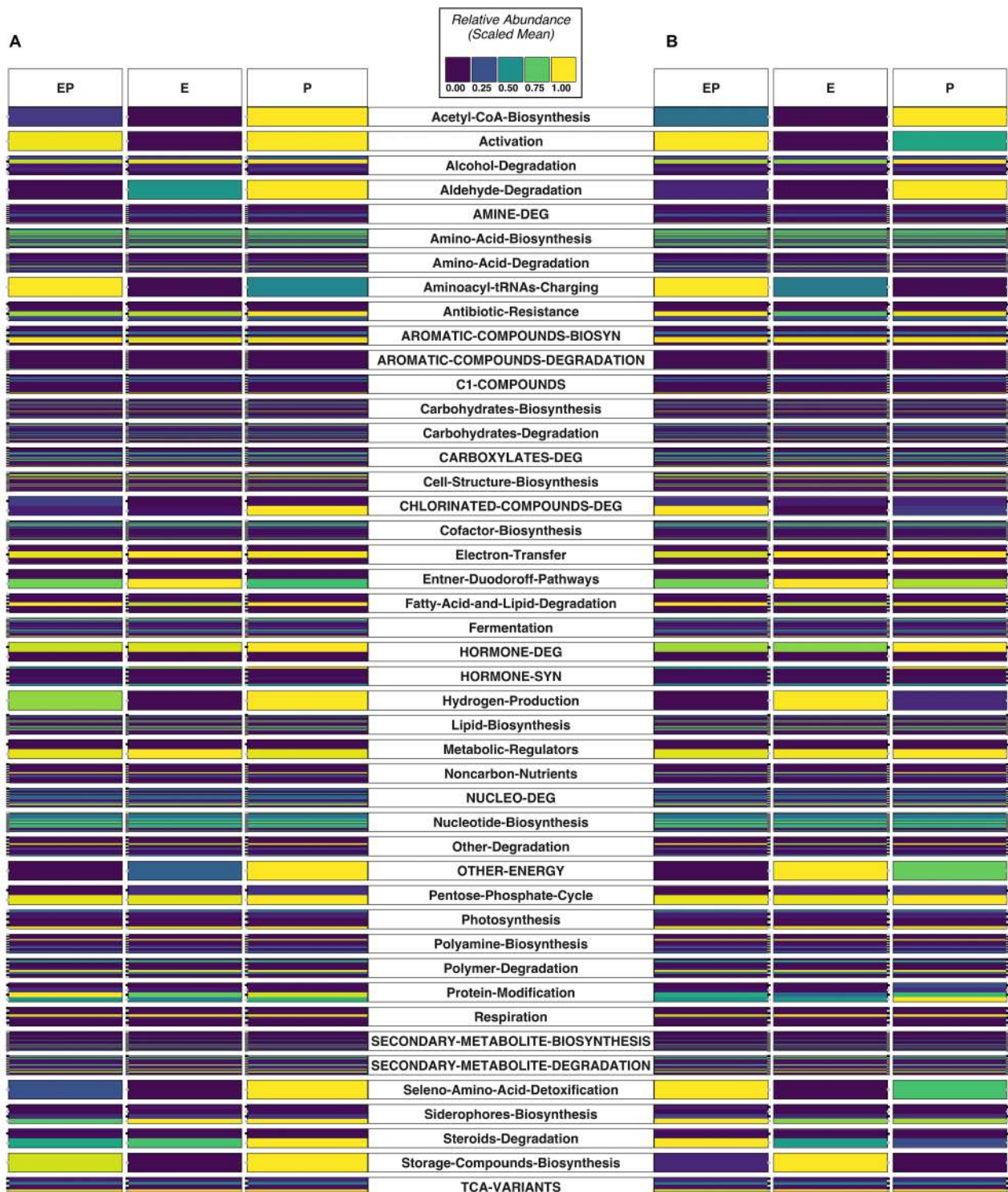

Supplement: FIG S4 [file sys003182228sf4.pdf]
