# Supplementary material for: A Prospective Metagenomic and Metabolomic Analysis of the Impact of Exercise and/or Whey Protein Supplementation on the Gut Microbiome of Sedentary Adults
Source: mSystems. 2018 Apr 24;3(3):e00044-18. doi: 10.1128/mSystems.00044-18 (PMC5915698; doi:10.1128/mSystems.00044-18)
Supplement: TABLE S5 [file sys003182228st5.docx]

|  | **Exercise (E) Group (n=25)** | **Exercise + Protein (EP) Group (n=22)** | **Protein only (P) Group**  **(n=27)** | **p-value** |
| --- | --- | --- | --- | --- |
| Interleukin 10 (pg/ml) | -0.05 (-0.21, 0.08) | 0 (-0.23, 0.19) | 0.02 (-0.11, 0.19) | 0.385 |
| Interleukin 6 (pg/ml) | -0.01 (-0.2, 0.2) | -0.04 (-0.28, 0.29) | 0 (-0.17, 0.23) | 0.893 |
| Interleukin 8 (pg/ml) | -5.53 (-29.79, 1.37) | -20.14  (-124.47, 0.61) ^Ψ^ | -1.63 (-3.56, 1.38) | 0.047* |
| TNF-α (pg/ml) | -0.1 (-0.66, 0.44) | -0.02 (-1.19, 0.77) | 0.11 (-0.24, 0.34) | 0.293 |
| IFN-γ (pg/ml) | 0.21 (-1.87, 3.39) | 0.07 (-1.72, 2.83) | -0.41 (-1.57, 1.3) | 0.962 |
| CRP (mg/L) | 0 (-0.5, 0) | 0 (0, 0) | 0 (0, 0) | 0.71 |
